# Social and ethical considerations in engaging American Indian and Alaska Native communities in HIV clinical research

**DOI:** 10.1186/1742-4690-9-S2-O26

**Published:** 2012-09-13

**Authors:** J Velcoff, DL Humes, R Foley, M Ignacio

**Affiliations:** 1 Fred Hutchinson Cancer Research Center, Seattle, WA, USA; 2 National Native American AIDS Prevention Center, Denver, CO, USA

## Background

American Indians and Alaska Natives (AI/AN) have the 4th highest rate of new HIV diagnoses among racial/ethnic groups and the highest mortality rate after an AIDS diagnosis (CDC, 2012), yet continue to be underrepresented in HIV clinical research trials due to historical and cultural factors. This presentation will highlight social and ethical experiences that contribute to mistrust of Western research and medicine and low involvement in HIV clinical research trials, and provide effective strategies for respectfully engaging Native communities.

The presentation will also describe the Native American Engagement in HIV Clinical Research (NAEHCR) project, a pilot aimed at increasing awareness and engagement in HIV clinical research with urban Indigenous communities using a participatory framework. NAEHCR was developed in partnership between the National Native American AIDS Prevention Center and the Legacy Project. NAEHCR is currently being conducted with DAIDS-funded research sites in Seattle, USA (HVTU, ACTU) and Denver, USA (HVTU, INSIGHT).

## Methods

Formative research assessed awareness, barriers, and facilitators of involvement in HIV clinical research using qualitative and quantitative methods. Focus groups were held with Native advisory boards to explore perceptions and experiences with clinical research. Individual interviews were conducted with clinical research site staff to assess perceptions and experiences with AI/AN communities. Surveys used to assess AI/AN community members’ awareness and experiences around clinical research.

## Results

Initial results indicate high levels of interest in the project among AI/AN community members and clinical research staff. Results also indicate low levels of awareness and engagement in HIV clinical research among the AI/AN community and a disconnect between the AI/AN community and HIV clinical research sites.

## Conclusion

Using a multi-method participatory approach offers a holistic depiction of barriers, opportunities for engagement, and important considerations when engaging AI/AN communities in HIV clinical research given the social and ethical legacy of research conducted in AI/AN communities.

